# Nutrition quality of life associated with affective functioning among Omani patients with type 2 diabetes from primary health care

**DOI:** 10.1017/jns.2020.57

**Published:** 2021-01-13

**Authors:** Masooma M. Al Toobi, Lyutha K. Al Subhi, Shekar Bose, Samir Al-Adawi

**Affiliations:** 1 Department of Food Science and Nutrition, College of Agricultural and Marine Sciences, Sultan Qaboos University, P. O. Box 34, Al Khoud 123, Oman; 2 Department of Natural Resource Economics, College of Agricultural and Marine Sciences, Sultan Qaboos University, P. O. Box 34, Al Khoud 123, Oman; 3 Department of Behavioral Medicine, College of Medicine and Health Sciences, Sultan Qaboos Muscat, P. O. Box 35, Al Khoud 123, Oman

**Keywords:** Nutrition quality of life, Anxiety and depression, Diabetes, Primary care, Affective functioning

## Abstract

Diabetes requires challenging lifelong dietary management, affects quality of life and heightens the impact of affective functioning. The aim of this study was to investigate the relationship between Nutrition Quality of Life (NQOL) and affective functioning in a sample of Omani patients with type 2 diabetes. A sample of 149 adults with type 2 diabetes was conveniently recruited from seven Primary Health Centers (PHCs) during follow-up visits. Data were gathered via face-to-face interviews. Pearson correlation and *χ*^2^ test of independence were applied to examine associations at *P* < 0⋅05. Most patients had poor glycemic control (71⋅1 %), BMI ≥ 25 kg/m^2^ (85⋅2 %) and central obesity (75⋅8 %), and moderate (54⋅4 %) and poor (32⋅9 %) level of NQOL. Based on the Hospital Anxiety and Depression Scale (HADS), 16⋅1 and 23⋅5 % of the sample endorsed the presence of anxiety and depression, respectively. A significant negative correlation was found between NQOL and HADS (*r* −0⋅590, *P* = 0⋅000), anxiety (*r* −0⋅597, *P* = 0⋅000) and depression (*r* −0⋅435, *P* = 0⋅000). There was a significant association between NQOL and HADS, *χ*^2^ (2) = 38⋅21, *P* < 0⋅01 that was large, Cramer's *V* = 0⋅51. Also, there were significant associations (*P* < 0⋅01) between NQOL and HADS when controlling for HbA1c, BMI, waist circumference and HMNT that were moderately to largely strong, Cramer's *V* = 0⋅43–0⋅55. There is an evident association between NQOL and affective functioning in adults with type 2 diabetes. Further research is recommended to confirm these relationships and to guide intervention programmes at PHCs to help improve the general quality of life of such patients.

## Introduction

Diabetes is endemic with an estimated worldwide incidence of 9⋅3 % in 2019 and is projected to reach 10⋅9 % by 2045. The Middle East and North Africa (MENA) region has the highest raw prevalence of diabetes (12⋅8 %) in 2019 and is further expected to rise to 15⋅7 % by 2045^(1)^. Diabetes is increasingly being recognised to be one of the medical conditions that require biopsychosocial approaches as poor Quality of Life (QOL) is a common fallout among those affected.

The WHO defines QOL as an ‘individual's perception of their position in life in the context of the culture and value systems in which they live and in relation to their goals, expectations, standards and concerns’ [p. 1]. QOL is an extensive concept influenced by intricate multi-personal and environmental factors^(2)^. A number of outcome-based measurements are in use to assess QOL in individuals with diabetes^(3)^. The progressive and debilitating nature of diabetes tends to adversely impact QOL. Studies reported significantly low QOL in individuals with diabetes^(4–7)^. In a comparison study of Canadians, the *Health-Related Quality of Life* (HR-QOL) was significantly lower in people with diabetes than others^(5)^. In an outpatient sample with type 2 diabetes (*n* 100), QOL was poorly perceived, as gauged by *Problem Area of Diabetes scale* (PAID)^(7)^.

Diabetes is a refractory condition that requires multifaceted, lifelong self-management for which individualised nutrition therapy is a cornerstone, irrespective of medical management^(8–11)^. However, dietary management is a challenge for various reasons^(10)^, though it positively impacts glycemic control and QOL when diabetes education interventions are provided^(12–14)^. Given the adverse impact of diabetes on QOL and the challenge of coping with day-to-day dietary management, assessing the effect of the latter on QOL would further guide the individualisation of patient care with respect to nutrition management. There is a dearth of such approaches assessing the trajectory of nutrition and QOL in the Eastern Mediterranean Region. An instrument known as *Nutrition Quality of Life* (NQOL) has been deemed to capture all domains representing the interface between nutrition and QOL^(15,16)^. NQOL specifically assesses food impact, self-image, psychological factors, social/interpersonal factors, physical functioning and self-efficacy that are not featured in other instruments designed to tap into QOL. While attempts have been made to quantify QOL among people with diabetes in Arabian Gulf countries, no studies were found that have specifically focused on NQOL. In non-Arabian countries, to date, only a few studies have been conducted on NQOL, of which two were on young healthy individuals. In one, the psychometric properties of NQOL were studied in relation to body mass index (BMI) and mindfulness^(17)^, while the other compared NQOL by socio-demographic characteristics of undergraduate students^(18)^. In a nursing home, NQOL significantly improved when elderly were given a liberalised diet plan for a period of 4 weeks moving from restricted diets^(19)^. In cancer patients, NQOL was significantly higher compared to others when provided with MNT^(20)^.

In addition to QOL, diabetes is one of the multimorbidity conditions that, without adequate self-management, often leads to various psychosocial dysfunctions^(21)^. The literature suggests that psychological burden is common in people with diabetes^(22)^ and within such background, there is a strong suggestion that there is an intimate link between diabetes and adverse moods such as depression and anxiety, or a mixture of them. One hypothesis suggests that affective disorders are likely to be precipitated by the onset of diabetes, but there is also a dissenting view^(23,24)^. Despite such a link, the recognition of mood disorders in people with diabetes has remained suboptimal because many studies have employed measures sensitive to psychological-cognitive symptoms rather than somatic-biological symptoms. The latter has been suggested to present the hallmark of affective disturbance in people with diabetes^(25)^. Few studies that are sensitive to somatic-biological symptoms among people with diabetes have been conducted in the Eastern Mediterranean region, i.e. Arabian Gulf countries, using measures^(26,27)^. The existing studies from the Arabian Gulf consolidate the view that diverse populations around with diabetes do display psychological burden; hence, more studies are therefore warranted. The purpose of this study is to investigate the following research questions: (1) Are there any statistically significant differences in the distribution of participants with reference to levels of NQOL, affective functioning, glycemic control, measures of obesity and history of medical nutrition therapy (HMNT)? (2) Are there any significant associations between NQOL and affective functioning and between each with other variables (glycemic control, measures of obesity and HMNT)? (3) Are there any statistically significant associations between the levels of NQOL and affective functioning controlling for glycemic control, measures of obesity and HMNT?

## Subjects and methods

This study was conducted according to the guidelines laid down in the Declaration of Helsinki and all procedures involving human subjects/patients were approved by the Central Research and Ethical Review and Approve Committee of the Ministry of Health (MH/DGP/R&S/PROPOSAL_APPROVED/4/2013) and the Regional Research and Ethics Committee of Governorate of Muscat (MH/DGHS/DPT/2/8/2013). Written informed consent was obtained from all subjects/patients.

### Study sample

Oman has universal healthcare coverage for its nationals and its healthcare system is divided into primary, secondary and tertiary care. This prospective, non-controlled study was conducted in Primary Health Centers (PHCs) in the urban area Governorate of Muscat, divided into six districts (Muscat, Muttrah, Bowshar, A'Seeb, Al Amerat and Quriyat)^(28)^, which had a total of twenty PHCs during the period of the study. The study included 1 % of the total number of patients registered with diabetes (*N* 14 909) in the Governorate in 2011^(29)^ which translated to 149 participants. The sample size was constrained by the availability of financial resources. In addition, the consideration of factors (based on the authors’ field experience) such as the likelihood of the respondent's motivation to participate in the interview due to time factor and the respondent's unwillingness to talk about their personal matters affected the determination of sample size for the present study.

As an initial step, a stratified sampling process based on the number of patients involved in the PHCs was applied in selecting the centres. Centres with ≤100 cases in their diabetes registry were excluded from sampling. PHCs were then randomly chosen by a number draw from each district. An exception was the district of A'Seeb from which two centres were selected due to its population density and the increased number of PHCs in the area^(28)^. The sample size from each selected health centre was calculated to be proportional to the number of diabetes cases recorded in its diabetes registry to match the overall sample size of the study (*n* 149). Only adult (19–65 years old), Omani Arabic-speaking individuals diagnosed with type 2 diabetes for ≥1 year were included. The study excluded non-Arabic-speaking Omani patients, persons <19 and >65 years old (the 65 years age limit was adapted to curb the number of illiterate individuals since the spread of education started in 1970 in Oman), persons on dialysis, pregnant females and persons having HbA1c values dated older than a year from the day of the interview. As a standard practice in PHCs, HbA1c is conducted on an annual basis for most of the patients.

#### Nutrition quality of life

The nutrition quality of life was gauged by *the Nutrition Quality of Life* (NQOL) questionnaire version 1.4. NQOL was developed by Barr and Schuacher to assess the impact of nutrition on QOL. This version of the NQOL consists of fifty items divided into six domains: food impact (nine items); self-image (six items); psychological factors (ten items); social/interpersonal factors (seven items); physical functioning (nine items); self-efficacy (nine items). All response options are on a five-point Likert scale (all of the time, most of the time, some of the time, little of the time and none of the time)^(15,16)^.

As an integral part of this study, NQOL was translated into Arabic. The translation was done following an established protocol^(30)^. In brief, the translated questionnaire was back-translated and revised by a language specialist and a nutrition specialist and modifications were implemented to clarify and simplify meanings accordingly. The questionnaire was then piloted with ten adults, graduate students and staff. They independently reviewed the items for ease of reading and clarity of meaning. A discussion was then conducted with a sub-group of five out of the ten adults for clarity and possible phrasings and wordings of some words due to linguistic variation among people from different regions in Muscat. The item ‘my food-related condition has caused problem with sexual relations’ was excluded for its socio-cultural misalignment with local religious and cultural beliefs as indicated by the pilot group.

The revised questionnaire was then tested on a convenient sample of nine patients with type 2 diabetes from two PHCs. Changes were adapted to simplify meanings for patients with low education levels, as emerged from this phase. In addition, the item ‘Liked the way my clothes fit’ was excluded as a result of criticism from patients that the item did not fall in line with what was considered to be culturally appropriate. In addition, the use of loose-fitting clothing among men and women is common in Oman. Thus, the final version of the adapted NQOL questionnaire consisted of forty-eight items. In the process, possible dialect synonyms and explanatory words were listed in case a need to clarify the meaning of phrases arose during the implementation.

Each item on NQOL was assessed on a scale of five points from 1 for ‘none of the time’ to 5 for ‘all of the time’ of the previous 2 weeks, with a minimum of 48 points and a maximum possible composite score of 240. For this study, NQOL scores were grouped into three levels: low (<75 %); moderate (75–89⋅9 %); high (≥90 %).

#### Affective functioning

The validated Arabic version of the *Hospital Anxiety and Depression Scale* (HADS) was used to screen for the presence of anxiety and depressive symptoms. The scale has fourteen screening items of which seven are for anxiety and the other seven items are for depression, assessed on a four-point Likert scale (0–3)^(31)^. The current scoring of HADS was based on the threshold of ≤7 on each subscale. For those who scored ≤7 are labelled as ‘non-case’ as set by the previous researchers^(31,32)^, while we labelled those scored >7 as ‘case-ness’. For brevity, this study collapsed three outcomes of HADS: HADS-Total (representing the sum score of the two subscales); HADS-Anxiety (denotes the sum of the items relevant to anxiety); HADS-Depression (denotes the sum of the items relevant to depression).

##### HbA1c, measures of obesity and history of medical nutrition therapy

The latest HbA1c values were abstracted from the medical records. As a standard practice, HbA1c investigation is only conducted once a year for most of the patients in PHC settings. Thus, only participating subjects with HbA1c dated within a year from the day of interviews were included. The WHO classification of glycemic control (HbA1c <7 %) was employed to denote adequate glycemic control^(33)^.

Three anthropometric measurements were taken in the vital rooms on the same day of the interview: height (cm); weight (kg); waist circumference (cm). Height and weight were used to calculate the BMI index of weight-for-height (kg/m^2^) and the latter was used to assess central obesity. Both BMI and waist circumference are recommended indices of adiposity classification for different purposes such as assessing the status or the need for intervention. The WHO cut-off points for classifications of BMI (18⋅5–24⋅9 kg/m^2^ (normal), 25–29⋅9 kg/m^2^ (overweight) and ≥30 kg/m^2^ (obesity))^(34)^ and sex-specific waist circumference (>102 cm for men and >88 cm for women) [p. 27]^(35)^ were applied.

MNT is recommended as a routine in the care for people with diabetes^(36)^; thus, it was deemed essential to explore whether the patient-seeking consultation from primary health care in Oman had been exposed to MNT and if it had an impact on their NQOL. In this study, MNT operationalised to constitute History of Medical Nutrition Therapy (HMNT) was assessed by the number of times participants reported encounters with nutritionists/dietitians since the diagnosis of diabetes. Reported counts of HMNT were then categorised as ‘0’, 1–3 and >3 times. Socio-demographic data, including age, gender, employment status, marital status, income and educational level, were also sought.

### Data collection

The present study was conducted in PHCs. In the stratified PHCs, patients showing up in the diabetes clinic for their follow-up were verbally invited to participate in the study. Patients who agreed to participate were asked to sign a consent form. Data were collected by three trained interviewers (nutritionist/dietitians). All interviewers were trained simultaneously on the tools. The interviewers were scrutinised for their consistency in dispensing the questionnaires. There was high concordance among them. Interviews were conducted either in the conference room of the PHC or in the waiting area depending on the participant's preference. Data were collected using online Google forms to minimise errors related to manual data entry. Data points on the electronic form were set as required to avoid any missing data. Seven identical electronic forms were created, one for each health centre to track the proportional number of patients specified for each centre and the progress of data collection.

### Statistical analyses

Statistical analysis was conducted using Statistical Package for the Social Science (SPSS). Demographic data were analysed by frequencies and percentages. The distribution of the continuous variables was assessed before analyses using the Jarque–Bera Lagrange multiplier (J-B LM) test that indicated that all the continuous variables were not normally distributed^(37)^. Cronbach's alpha (with 95 % CI) was applied to assess the size of the internal reliability of the NQOL and HADS. The Pearson's product moment correlation coefficient was used to measure the relationships between the NQOL and HADS as all the concerned variables are continues in nature. A non-parametric statistical test (i.e. the *χ*^2^ test of independence) on two- and three-way contingency tables was conducted to check the association between groups of variables of interests (i.e. NQOL and HADS with other variables (HADS-Total, anxiety, depression, gender, HbA1c, BMI, waist circumference, HMNT, etc.)). The *χ*^2^ test of independence determines the statistical significance of the association between variables but not the extent of such relationships. Therefore, Cramér's *V* coefficient (also known as Cramér's phi) which uses *χ*^2^ statistic was calculated to examine the strength of the relationship between variables. The value of the Cramér's *V* coefficient (CV) ranges from 0 to 1. *A priori P* < 0⋅05 was considered significant.

## Results

Both scales, NQOL and HADS, had high levels of internal consistency, as determined by a Cronbach's alpha of 0⋅880 (95 % CI 0⋅85, 0⋅91) and 0⋅863 (95 % CI 0⋅83, 0⋅89), respectively.

### Characteristics of the study participants

The characteristics and the distribution of the study participants are presented in Tables 1 and 2. With mostly females and low-income individuals, a large proportion of the study sample is classified as overweight (38⋅9 %) and obese (46⋅3 %). Most of the participants had central obesity (75⋅8 %) and poor glycemic control (71⋅1 %) as noted by HbA1c ≥ 7.

**Table 1.** Socio-economic profile of Omani patients with type 2 diabetes seeking care in primary health centres in Muscat

Variables	*n*	%
Gender		
Male	45	30⋅2
Female	104	69⋅8
Employment		
Employed	44	29⋅5
Unemployed	86	57⋅7
Retired	19	12⋅8
Marital status		
Unmarried	35	23⋅5
Married	114	76⋅5
Income (OMR)		
≤400	78	52⋅3
401–800	50	33⋅6
≥801	21	14⋅1
Education		
≤No formal education	76	51⋅0
Less than secondary school	32	21⋅5
Secondary	22	14⋅8
Post-Secondary	19	12⋅7

OMR (Omani Rial). 1 OMR ≈ USD 2⋅59 (pegged).

**Table 2.** Distribution of the participants with type 2 diabetes by the study variables and *χ*^2^ test results

Variables	%	Observed frequency	Expected frequency	*χ*^2^ (d.f.)	*P*-value*
NQOL^a^				38⋅7 (2)	<0⋅001
Low (<75 %)	32⋅9	49	49⋅7		
Moderate (75–89⋅9 %)	54⋅4	81	49⋅7		
High (≥90 %)	12⋅7	19	49⋅7		
HADS-Total^b^				63⋅1 (1)	<0⋅001
Non-case (≤14)	82⋅5	123	74⋅5		
Case-ness (>14)	17⋅5	26	74⋅5		
HADS-Anxiety^b^				68⋅5 (1)	<0⋅001
Non-case (≤7)	83⋅9	125	74⋅5		
Case-ness (>7)	16⋅1	24	74⋅5		
HADS-Depression^b^				41⋅9 (1)	<0⋅001
Non-case (≤7)	76⋅5	114	74⋅5		
Case-ness (>7)	23⋅5	35	74⋅5		
HbA1c (%)^c^				26⋅6 (1)	<0⋅001
<7	28⋅9	43	74⋅5		
≥7	71⋅1	106	74⋅5		
BMI (kg/m^2^)^d^				24⋅3 (2)	<0⋅001
18⋅5–24⋅9	14⋅8	22	49⋅7		
25-29⋅9	38⋅9	58	49⋅7		
≥30	46⋅3	69	49⋅7		
Waist Circumference (cm): Males				0⋅2 (1)	0⋅655
≤102	16⋅1	24	22⋅5		
>102	14⋅1	21	22⋅5		
Waist Circumference (cm): Females				61⋅5 (1)	<0⋅001
≤88	08⋅1	12	52		
>88	61⋅7	92	52		
HMNT^e^				70⋅2 (2)	<0⋅001
0 times	10⋅1	15	49⋅7		
1–3 times	64⋅4	96	49⋅7		
>3 times	25⋅5	38	49⋅7		

d.f., degree of freedom.

a Nutrition Quality of Life (NQOL).

b Hospital Anxiety and Depression Scale (HADS)-Total based on the sum of all subscales of HADS (i.e. HADS-Anxiety and HADS-Depression).

c HbA1c refers to glycated haemoglobin.

d Body mass index (BMI) weight-for-height (kg/m^2^).

e History of Medical Nutrition Therapy (HMNT) represents the number of times subjects reported to have received MNT.

* *P*-value < 0⋅05 indicates statistical significance at the 5 % level.

A significant difference in the distribution of participants among the three levels of NQOL, *χ*^2^ (2) 38⋅71, *P* < 0⋅001, was observed in all variables but waist circumference in males. Much of the sample had reported having 1–3 encounters with a dietitian (Table 2).

### NQOL and HADS associations

The relationship between NQOL and HADS was found to be negative and significant. Significant positive correlations were found between HADS score and its subscales, anxiety (*r* 0⋅885, *P* < 0⋅001) and depression (*r* 0⋅871, *P* < 0⋅001) (Table 3). Results of the two-way *χ*^2^ test of independence are presented in Table 4. There was a significant association between levels of NQOL with levels of HADS-Total (*χ*^2^ (2) = 38⋅2, *P* < 0⋅001), anxiety (*χ*^2^ (2) = 38⋅7, *P* < 0⋅001) and depression (*χ*^2^ (2) = 22⋅3, *P* < 0⋅001), with associations from highly to moderately strong^(38)^, Cramér's *V* (CV) = 0⋅51, 0⋅51 and 0⋅39, respectively.

**Table 3.** Correlations between nutrition quality of life and hospital anxiety and depression scale, and with other variables

Variables	NQOL^a^		HADS^b^	
	Correlation coefficient (*r*)	*P*-value*	Correlation coefficient (*r*)	*P*-value*
HADS-Total	−0⋅590	<0⋅001	NA	NA
HADS-Anxiety	−0⋅597	<0⋅001	0⋅885	<0⋅001
HADS-Depression	−0⋅435	<0⋅001	0⋅871	<0⋅001
HbA1c (%)^c^	0⋅086	0⋅297	−0⋅035	0⋅672
BMI (kg/m^2^)^d^	−0⋅156	0⋅058	0⋅119	0⋅147
Waist Circumference (cm)	−0⋅068	0⋅408	0⋅114	0⋅164
HMNT^e^	0⋅002	0⋅984	−0⋅023	0⋅782
Age (years)	0⋅239	0⋅003	−0⋅081	0⋅323

NA, not applicable.

a Nutrition Quality of life (NQOL).

b Hospital Anxiety and Depression Scale (HADS).

c HbA1c refers to glycated haemoglobin.

d Body mass index (BMI) weight-for-height (kg/m^2^).

e History of Medical Nutrition Therapy (HMNT).

* *P*-value < 0⋅05 indicates the statistical significance of the associated coefficient at the 5 % level.

**Table 4.** The association between nutrition quality of life and affective functioning and with other variables

Variables	NQOL^a^		HADS^b^	
	*χ*^2^ (d.f.)	*P*-value (CV)^f^	*χ*^2^ (d.f.)	*P*-value*
HADS-Total	38⋅2 (2)	<0⋅001 (0⋅51)	NA	NA
Non-case				
Otherwise				
HADS-Anxiety	38⋅7 (2)	<0⋅001 (0⋅51)	NA	NA
Non-case				
Case-ness				
HADS-Depression	22⋅4 (2)	<0⋅001 (0⋅39)	NA	NA
Non-case				
Case-ness				
Gender	1⋅6 (2)	0⋅46	1⋅0 (1)	0⋅31
Male				
Female				
HbA1c (%)^c^	1⋅9 (2)	0⋅38	0⋅5 (1)	0⋅48
<7				
≥7				
BMI (kg/m^2^)^d^	6⋅3 (4)	0⋅18	2⋅7 (2)	0⋅25
Normal				
Overweight				
Obese				
Waist Circumference (cm): Male	2⋅1 (2)	0⋅36	0⋅1 (1)	0⋅81
Desirable (≤ 102 cm)				
Undesirable (>120 cm)				
Waist Circumference (cm): Female	1⋅8 (2)	0⋅41	0⋅1 (1)	0⋅33
Desirable (≤88 cm)				
Undesirable (>88 cm)				
HMNT^e^	4⋅4 (2)	0⋅11	1⋅3 (1)	0⋅25
0 time				
≥1 time				
HMNT	3⋅2 (2)	0⋅20	0⋅3 (1)	0⋅59
1–3 times				
>3 times				

d.f., degree of freedom; NA, not applicable.

a Nutrition Quality of life (NQOL).

b Hospital Anxiety and Depression Scale (HADS).

c HbA1c refers to glycated haemoglobin.

d Body mass index (BMI) weight-for-height (kg/m^2^).

e History of Medical Nutrition Therapy (HMNT).

f Cramér's *V* coefficient (CV) estimate is in the parentheses. Reported only for statistically significant (at least at the 5 % level) cases.

* *P*-value < 0⋅05 indicates statistical significance at the 5 % level.

The results of the three-way *χ*^2^ test of independence are presented in Table 5. It is evident that in participants with HbA1c ≥7 %, there are significant associations between NQOL with all measures of HADS: HADS-Total, *χ*^2^ (2) = 19⋅42, *P* < 0⋅001; HADS-Anxiety, *χ*^2^ (2) = 27⋅62, *P* < 0⋅001; HADS-Depression, *χ*^2^ (2) = 8⋅85, *P* 0⋅01. The associations were high to moderately strong^(38)^, Cramér's *V* (CV) = 0⋅43, 0⋅51 and 0⋅29, respectively. Significant associations (*P* < 0⋅001) are observed in all *χ*^2^ testes that meet the assumption of test, BMI ≥ 25 kg/m^2^, undesirable waist circumference, HMNT ≥ 1 and one to three times of HNMT.

**Table 5.** The association between nutrition quality of life and the hospital anxiety depression scale and other variables

Variables	NQOL^a^ and HADS^b^-Total		NQOL and HADS-Anxiety		NQOL and HADS-Depression	
	*χ*^2^ (d.f.)	*P*-value* (CV)^c^	*χ*^2^ (d.f.)	*P*-value* (CV)^c^	*χ*^2^ (d.f.)	*P*-value* (CV)^c^
HbA1c (%)^d^						
<7	19⋅2 (2)	<0⋅001 (0⋅67)	11⋅8 (2)	0⋅003 (0⋅52)	15⋅2 (2)	<0⋅001 (0⋅60)
≥7	19⋅4 (2)	<0⋅001 (0⋅43)	27⋅6 (2)	<0⋅001 (0⋅51)	8⋅8 (2)	0⋅012 (0⋅29)
BMI (kg/m^2^)^e^						
<25	10⋅1 (2)	0⋅006 (0⋅68)	10⋅5 (2)	0⋅005 (0⋅69)	6⋅2 (2)	0⋅045 (0⋅53)
≥25	29⋅0 (2)	<0⋅001 (0⋅48)	29⋅0 (2)	<0⋅001 (0⋅48)	20⋅0 (2)	<0⋅001 (0⋅40)
Aggregate Waist Circumference (cm)^f^						
Desirable	6⋅0 (2)	0⋅510	8⋅1 (2)	0⋅017 (0⋅47)	6⋅1 (2)	0⋅048 (0⋅41)
Undesirable	33⋅9 (2)	<0⋅001 (0⋅55)	31⋅0 (2)	<0⋅001 (0⋅52)	17⋅3 (2)	<0⋅001 (0⋅39)
HMNT^g^						
0 time	7⋅0 (2)	0⋅031 (0⋅68)	NA	NA	2⋅7 (2)	0⋅254
≥1 time	32⋅3 (2)	<0⋅001 (0⋅49)	35⋅4 (2)	<0⋅001 (0⋅51)	19⋅2 (2)	<0⋅001 (0⋅38)
HMNT						
1–3 times	17⋅4 (2)	<0⋅001 (0⋅43)	22⋅1 (2)	<0⋅001 (0⋅48)	13⋅4 (2)	0⋅001 (0⋅37)
>3 times	17⋅5 (2)	<0⋅001 (0⋅68)	14⋅1 (2)	0⋅001 (0⋅61)	5⋅8 (2)	0⋅055

d.f., degree of freedom; NA, not applicable.

a Nutrition Quality of life (NQOL).

b Hospital Anxiety and Depression Scale (HADS).

c Cramér's *V* coefficient (CV) estimate is in the parentheses. Reported only for statistically significant (at least at the 5 % level) cases.

d HbA1c refers to glycated haemoglobin.

e Body mass index (BMI) weight-for-height (kg/m^2^).

f Aggregated waist circumference groups (Desirable: ≤102 cm (male) and ≤88 cm (female); Undesirable: >102 cm (male) and >88 cm (female)).

g History of Medical Nutrition Therapy (HMNT).

* *P*-value < 0⋅05 indicates statistical significance at the 5 % level.

## Discussion

With our sample of type 2 diabetes, NQOL and HADS showed high internal reliability (*α* = 0⋅88 and *α* = 0⋅86, respectively) with tight ranges of 95 % CI signifying the size of reliability^(39)^. Both alpha levels are higher than the satisfactory minimum reliability (*α* = 0⋅7) suggested by Nunnally and Bernstein^(40)^. Also, the reliability of the NQOL (48-item) in our sample is higher than those reported on the 50-item NQOL surveys of American (*α* = 0⋅74)^(17)^ and Malay (*α* = 0⋅217–0⋅908; *n* 241) undergraduate students^(18)^. Although the NQOL scale is intended to gauge nutrition-related quality of life of individuals receiving MNT^(15,16)^ such as those in our sample, studies on NQOL and MNT^(20,41,42)^ did not report psychometric properties of the instrument.

As in the present study, the NQOL cut-off scores are set arbitrarily due to limited research in the field. In this study, NQOL score ≥90 % was deemed high and <75 % was deemed low. In cancer patients, a score of ≥76 % is set as acceptable^(20)^ based on a dichotomous NQOL scale (agree, disagree) while we used a 5-point Likert scale. Other studies reported means of NQOL. On a 47-item NQOL survey, a mean score of 66 % ± 10 is reported for post-gastric bypass surgery individuals (*n* 72)^(42)^, while a higher mean score (145⋅8 ± 23⋅1) is reported in college students (*n* 182)^(17)^ using a 50-item NQOL survey; however, students form a special population. Wampler only included five items of the NQOL survey from two constructs (social/interpersonal and psychological)^(41)^, and hence, it is not comparable to our findings. Differences in the number of items and the cut-off points of NQOL are evident among studies and comparison should be considered with caution.

Various measures have been employed to tap into mood symptoms of people with diabetes. It is increasingly being recognised that questionnaires soliciting somatic-biological symptoms rather than those soliciting psychological-cognitive symptoms are more likely to capture phenotypical presentation of mood disturbance in people with diabetes^(25)^. Studies have suggested that HADS is more equipped to tap into somatic-biological symptoms since it was initially described for use among people with medical conditions rather than psychiatric disorders^(25)^. When correlated, both the aggregated and segregated HADS scores were correlated negatively and significantly with NQOL. To our knowledge, this is the first study that explores the variation of NQOL and mood symptoms.

A 17⋅5 % of our patients endorsed themselves to be having mood symptoms (HADS-Total) which outshines the 9⋅3 % reported in a large (*n* 245 404 of which 2 % had diabetes) multination study by WHO^(43)^. Also, a lower prevalence of both anxiety (9 %) and depression (20⋅9 %) was found in individuals with type 1 and type 2 diabetes in Italy^(44)^ compared with current 16⋅1 and 23⋅5 % endorsing case-ness on the subscales HADS-Anxiety and HADS-Depression, respectively. The incidence of anxiety in this sample is proximal to that found (15⋅6 %) in a large multi-racial (*n* 20 142) American study on the coexistence of anxiety and diabetes conducted based on the PHQ-8 scale^(45)^.

Differences in the prevalence of mood symptoms are seen in studies from the same region as well. Depression in our sample with diabetes is higher than that of a diverse large sample (8⋅1 %; *n* 2005) attending PHCs in Muscat Governorate as assess by PHQ-9^(46)^. On the other hand, our prevalence appears to echo that reported in subjects with diabetes in Jordan where mood symptoms, based on the PHQ-8 scale, amounted to 19⋅7 % (*n* 649)^(47)^. On the other hand, the prevalence of mood symptoms in our study is considered high compared to that (12⋅5 %) from the United Arab Emirates (UAE) for subjects with diabetes from PHC (*n* 347), based on the *Kessler Psychological Distress* Scale (K6)^(26)^. In contrast, the prevalence in this study is lower than that reported in Qatar where anxiety was prevalent among 73 % and depression among 52⋅5 % in people with diabetes (*n* 889), assessed by the *Depression, Anxiety and Stress Scale* (DASS-21)^(27)^.

The participants in this study have a high average HbA1c (8⋅3 % ± 2) and the most (71 %) have poor glycemic control, and such a trend is commonly reported among people with diabetes in both developed and developing nations^(48–50)^. The American Diabetes Association (ADA) noted that poor glycemic control is common in nearly 50 % of people with diabetes^(10)^. The current sample with diabetes has increased measures of adiposity which echoes the general trend in Oman where the prevalence of overweight and obesity ranges from 46 %^(51)^ to 65⋅5 %^(52)^. With such high proportions of adiposity, weight management interventions are warranted for patients with type 2 diabetes in PHC to ameliorate HbA1c and blood lipids to curb diabetes complications^(14)^.

Although most participants (89⋅9 %) received at least one encounter of MNT with a dietitian with the majority (64⋅4 %) reporting 1–3 encounters, yet the majority have high measures of adiposity and poor glycemic control. More encounters with dietitians and evaluation of MNT services appear necessary to boost MNT for diabetes management in PHCs. Based on the Evidence Analyses Library (EAL), at least 3–6 encounters of MNT during the first 6 months of diagnosis are recommended^(53)^. For many, determining what and how much to eat are challenging while implementing diabetes management plans and there is not a ‘one-size-fits-all’ eating pattern. Thus, the ADA recommends that people with diabetes be actively and collaboratively engaged in the development of individualised eating plans^(10)^. Also, attention is due to provide literacy-appropriate MNT approaches, since the vast majority of the participants in our study did not obtain formal education and were of low income, in order to improve diabetes control in low-income groups^(54)^.

Since there are no studies that have investigated NQOL in people with diabetes, findings of this study are compared to both, findings on NQOL as available and studies on QOL in people with diabetes. A negative correlation between NQOL and affective functioning (HADS-Total) (*r* −0⋅59; *P* < 0⋅001) was expected. As diabetes management requires dietary changes that are challenging to those affected^(10)^, depression and anxiety could precipitate as a result and/or *vice versa*. This relationship guides us to conclude that NQOL is a practical tool to assess the impact of diabetes on NQOL and guide the direction of MNT in PHCs. In establishing criteria-related validity, NQOL was positively correlated with both, perceived health status as assessed by the Short-Form Health Survey (SF-36) (*r* 0⋅57; *P* < 0⋅001) and mindfulness (*r* 0⋅46; *P* < 0⋅001) based on Mindful Attention Awareness Scale (MAAS) among healthy students^(17)^. Also, similar correlations are reported between various scales of QOL and depression and anxiety among people with diabetes. QOL, based on the PAID scale, correlated with depressive symptoms based on the BDI scale (*r* 0⋅503; *P* < 0⋅001) in patients (*n* 100) with type 2 diabetes^(7)^. Paschalides *et al*. reported significant correlations between QOL and anxiety and depression, using the SF-36 for health-related QOL and WBQ scale for anxiety and depression in adult outpatients with type 2 diabetes (*n* 184)^(55)^.

Lifelong dietary management is a cornerstone for diabetes management and it has a positive effect on glycemic control^(56–59)^ and QOL^(12,13)^. However, no significant correlation was found between NQOL and HbA1c in the present study, which could partly be attributed to the disproportionally large number of patients had HbA1c ≥7 % and less than the recommended encounters of MNT. It is also possible that NQOL is designed to measure nutritional habits in the last 2 weeks, while the HbA1c values in this study were within a year from the day of the interview. Thus, variable findings could be revealed using recent HbA1c readings. Others also did not find significant correlations between HbA1c and QOL^(7)^ or depression and anxiety^(55)^ though low depression scores, using HADS, were significantly associated with good glycemic control (*P* < 0⋅01) in a large sample (*n* 1456) of Irish people with diabetes^(60)^. Such a difference could be attributed to the multifold difference in sample sizes, demographic differences and analyses approach.

The moderate to large^(38)^ significant associations between levels NQOL and HADS (Total, anxiety and depression) are in line with the significant negative correlation between NQOL and affective functioning (HADS) in our patients, and as reported by others between QOL and anxiety and depression^(7,55)^. No significant associations were found between NQOL and other variables in this sample, while healthy Malay female students exhibited a general tendency for higher NQOL than males^(18)^, and provision of MNT positively influenced NQOL in cancer patients (*n* 12)^(20)^ and nursing home residents (*n* 30)^(19)^, and QOL in adults with type 2 diabetes^(13,14)^. The present study used reported a history of encounters of MNT which could be negatively influenced by recall bias and the low level of education among the majority (>72 %) of the sample. With that, it is imperative to investigate the quality and approaches of MNT practices in diabetes clinics in PHCs in Muscat to gain a better insight on their impact on the recipients of the services. As for HADS, no significant associations were observed with other variables, while different studies reported that depression is significantly higher in females^(47,61,62)^.

In the three-way analyses, poor glycemic control, BMI ≥ 25 kg/m^2^, and central obesity are contributing factors to the significant association between NQOL and affective functioning in our sample and are also linked to affective functioning in relation to diabetes elsewhere^(7,63)^. In addition, the contribution of the history of MNT to the significant association between NQOL and affective functioning is also in line with the positive associations reported in intervention studies between MNT and QOL^(13,14)^, significant positive interactions between QOL and affective functioning^(4,64)^ and the significant interactions among QOL, depressive symptoms and glycemic control^(7)^ in persons with diabetes. With such findings and as noted earlier a significant negative correlation (*r* −0⋅59, *P* < 0⋅1) is in place in our sample, MNT appears important to enhance the NQOL that in turn would reflect on reduced levels of anxiety and depression.

### Conclusions and recommendations

Overall, there is a large negative correlation between NQOL and affective functioning. With categorical testing, the two variables also exhibited large association. When controlled for other variables, the association between them persisted though the effect size was marginally decreased due to sample size. Examination of the sensitivity of the outcome variable (NQOL) to potential predictor variables using logistic regression techniques should also be a useful addition to the present research. The incidence of anxiety and depression is higher than in other studies and is coupled with high rates of poor measures of obesity and HbA1c. Hence, preventative actions tackling anxiety and depression and lifestyle modification interventions should be considered in people with diabetes at PHC services in Oman. The Arabic NQOL survey (available on request) exhibited large internal reliability and further studies are recommended to validate the measure among other Arabian populations since it is a new tool and had not been widely established.

### Limitations

There are some shortcomings in the study to be acknowledged. The study is based on a small sample from one region of the country. Thus, any attempt to generalise the findings should be made with caution. During translation to Arabic, two of the items on the NQOL 1.4 were deemed as being unadaptable to socio-cultural norms in Oman. Such omission would ostensibly compromise the present findings compared to those that have employed the full version of NQOL version 1.4. Finally, the study relied on existing data of HbA1c as permitted by the standard practice in PHCs. Hence, the correlation between NQOL and HAD with HbA1c might be different if HbA1c readings were standardised.

## References

[1] International Diabetes Federation (2019) IDF Diabetes Atlas, 9th ed. International Diabetes Federation. https://www.diabetesatlas.org/upload/resources/2019/IDF_Atlas_9th_Edition_2019.pdf (accessed 15 October 2020

[2] World Health Organization (1997) WHOQOL Measuring Quality of Life. http://www.who.int/mental_health/media/68.pdf (accessed June 2019).

[3] Rubin RR & Peyrot M (1999) Quality of life and diabetes. Diabetes Metab Res Rev 15, 205–218. 10441043 10.1002/(sici)1520-7560(199905/06)15:3<205::aid-dmrr29>3.0.co;2-o

[4] Al-Maskari M, Petrini K, Al-Zakwani I, (2011) Mood dysfunction and health-related quality of life among type-2 diabetes in Oman: preliminary study. J Affect Disord 1, 56–63.

[5] Sikdar KC, Wang PP, MacDonald D, (2010) Diabetes and its impact on health-related quality of life: a life table analysis. Qual Life Res 19, 781–787. 20349211 10.1007/s11136-010-9641-5

[6] Rwegerera GM, Moshomo T, Gaenamong M, (2018) Health-related quality of life and associated factors among patients with diabetes mellitus in Botswana. Alex J Med 54, 111–118.

[7] Papelbaum M, Lemos HM, Duchesne M, (2010) The association between quality of life, depressive symptoms and glycemic control in a group of type 2 diabetes patients. Diabetes Res Clin Pract Suppl 89, 227–230. 10.1016/j.diabres.2010.05.024 20696361

[8] American Diabetes Association (2018) Standards of Medical Care in Diabetes—2018. Diabetes Care 41, S1.29222369

[9] Gray A & Threlkeld RJ (2015) Nutritional Recommendations for Individuals with Diabetes. [Updated 2019 Oct 13]. In Endotext [Internet], Feingold KR, Anawalt B, Boyce A Eds., South Dartmouth, MA: MDText.com, Inc.

[10] American Diabetes Association (2015) Foundations of care: education, nutrition, physical activity, smoking cessation, psychosocial care, and immunization. Diabetes Care 38, S20. 25537702 10.2337/dc15-S007

[11] Davies MJ, D'Alessio DA, Fradkin J, (2018) Management of hyperglycaemia in type 2 diabetes, 2018. A consensus report by the American Diabetes Association (ADA) and the European Association for the Study of Diabetes (EASD). Diabetologia 61, 2461–2498. 30288571 10.1007/s00125-018-4729-5

[12] Chrvala CA, Sherr D & Lipman RD (2016) Diabetes self-management education for adults with type 2 diabetes mellitus: a systematic review of the effect on glycemic control. Patient Educ Couns 99, 926–943. 26658704 10.1016/j.pec.2015.11.003

[13] Lemon CC, Lacey K, Lohse B, (2004) Outcomes monitoring of health, behavior, and quality of life after nutrition intervention in adults with type 2 diabetes. J Am Diet Assoc 104, 1805–1815. 15565074 10.1016/j.jada.2004.09.024

[14] Wolf AM, Conaway MR, Crowther JQ, (2004) Translating lifestyle intervention to practice in obese patients with type 2 diabetes – improving control with activity and nutrition (ICAN) study. Diabetes Care 27, 1570–1576. 15220230 10.2337/diacare.27.7.1570

[15] Barr J & Schumacher G (2003) Using focus groups to determine what constitutes quality of life in clients receiving medical nutrition therapy: first steps in the development of a nutrition quality-of-life survey. J Am Diet Assoc 103, 844–851. 12830022 10.1016/s0002-8223(03)00385-7

[16] Barr J & Schumacher G (2003) The need for a nutrition-related quality-of-life measure. J Am Diet Assoc 103, 177–180. 12589322 10.1053/jada.2003.50058

[17] Cochran CR (2010) Nutrition quality of life : correlations of the NQOL, body mass index, and mindfulness. Masters Thesis, University of South Alabama. ProQuest Dissertations and Theses (PQDT).

[18] Pei Lin L, Wan Putri Elena WD & Mohd Razif S (2012) Nutrition quality of life among female-majority Malay undergraduate students of health sciences. Malays J Med Sci 19, 37–49. 23785251 PMC3684233

[19] Tanner EM (2011) Examination of liberalized diets for nursing home residents and their impact on dietary intake and nutrition quality of life. M.Sc Thesis, D'Youville College.

[20] McCarthy MA (2009) Perceived quality of life of cancer patients. 1471696 M.S., D'Youville College.

[21] Bolton D & Gillett G (2019) The biopsychosocial model 40 years on. In The Biopsychosocial Model of Health and Disease: New Philosophical and Scientific Developments, pp. 1–43 [D Bolton and G Gillett, editors]. Cham: Springer International Publishing. 31886965

[22] Stoop C, Pouwer F, Pop V, (2019) Psychosocial health care needs of people with type 2 diabetes in primary care: views of patients and health care providers. J Adv Nurs 2019, 1–11. 10.1111/jan.13996 PMC6850404 30883846

[23] Lustman PJ & Clouse RE (2007) Depression in diabetes: the chicken or the egg? Psychosom Med 69, 297–299. 17517972 10.1097/PSY.0b013e318060cc2d

[24] Renn BN, Feliciano L & Segal DL (2011) The bidirectional relationship of depression and diabetes: a systematic review. Clin Psychol Rev 31, 1239–1246. 21963669 10.1016/j.cpr.2011.08.001

[25] Nicolau J, Simo R, Conchillo C, (2019) Differences in the cluster of depressive symptoms between subjects with type 2 diabetes and individuals with a major depressive disorder and without diabetes. J Endocrinol Invest 42, 881–888. 30788770 10.1007/s40618-019-01020-x

[26] Sulaiman N, Hamdan A, Tamim H, (2010) The prevalence and correlates of depression and anxiety in a sample of diabetic patients in Sharjah, United Arab Emirates. BMC Fam Pract 11, 1–7. 20973956 10.1186/1471-2296-11-80 PMC2987911

[27] Bener A, Al-Hamaq A & Dafeeah EE (2011) High prevalence of depression, anxiety and stress symptoms among diabetes mellitus patients. Open Psychiatr Journal 5, 5–12.

[28] Municipal Council Muscat (2019) Wilayats in Muscat and Its Representative. https://www.mmc.gov.om/wilayat.aspx (accessed March 2019).

[29] Ministry of Health (2012) Annual Health Report. http://www.moh.gov.om/en/stat/2011/index_eng.htm (accessed January 2014).

[30] Sartorius N & Kuyken W (1994) Translation of Health Status Instruments, Quality of Life Assessment: International Perspectives. Berlin, Heidelberg: Springer Berlin Heidelberg.

[31] El-Rufaie OE & Absood G (1987) Validity study of the hospital anxiety and depression scale among a group of Saudi patients. Br J Psychiatry 151, 687–688. 3446314 10.1192/bjp.151.5.687

[32] Zigmond AS & Snaith RP (1983) The hospital anxiety and depression scale. Acta Psychiatr Scand 67, 361–370. 6880820 10.1111/j.1600-0447.1983.tb09716.x

[33] World Health Organization (2011) Use of glycated haemoglobin (HbA1c) in diagnosis of diabetes mellitus: abbreviated report of a WHO consultation. World Health Organization. https://apps.who.int/iris/handle/10665/70523 (accessed 5 June 2019). 26158184

[34] WHO Expert Consultation (2004) Appropriate body-mass index for Asian populations and its implications for policy and intervention strategies. Lancet 363, 157–163. 14726171 10.1016/S0140-6736(03)15268-3

[35] World Health Organization (2011) Waist Circumference and Waist-Hip Ratio: Report of a WHO Expert Consultation. Geneva, 8–11 December 2008. https://www.who.int/nutrition/publications/obesity/WHO_report_waistcircumference_and_waisthip_ratio/en/ (accessed January 2012).

[36] American Diabetes Association (2015) Standards of medical care in diabetes – 2015 abridged for primary care providers. Clin Diabetes 33, 97–111. 25897193 10.2337/diaclin.33.2.97 PMC4398006

[37] Jarque CM & Bera AK (1980) Efficient tests for normality, homoscedasticity and serial independence of regression residuals. Econ Lett 6, 255–259.

[38] Cohen J (1988) Statistical power analysis for the behavioral sciences, 2nd ed. Hillsdale, NJ: Lawrence Erlbaum Associates.

[39] Iacobucci D & Duhachek A (2003) Advancing alpha: measuring reliability with confidence. J Consum Psychol 13, 478–487.

[40] Nunnally JC & Bernstein IH (1994) Psychometric Theory, 3rd ed. New York: McGraw-Hill.

[41] Wampler AL (2006) Gastrointestinal Symptoms, Measures of Adiposity, and Diet and Lifestyle Practices in Patients with Gastroesophageal Reflux Disease and Matched Controls. Master of Science, University of Kansas.

[42] Hunt AE, Wilson RM, Pope JF, (2007) Nutrition-related quality of life after laparoscopic gastric bypass. Top Clin Nutr 22, 156–161.

[43] Moussavi S, Chatterji S, Verdes E, (2007) Depression, chronic diseases, and decrements in health: results from the World Health Surveys. Lancet 370, 851–858. 17826170 10.1016/S0140-6736(07)61415-9

[44] Trento M, Raballo M, Trevisan M, (2012) A cross-sectional survey of depression, anxiety, and cognitive function in patients with type 2 diabetes. Acta Diabetol 49, 199–203. 21442429 10.1007/s00592-011-0275-z

[45] Li C, Barker L, Ford ES, (2008) Diabetes and anxiety in US adults: findings from the 2006 Behavioral Risk Factor Surveillance System. Diabet Med 25, 878–881. 18644077 10.1111/j.1464-5491.2008.02477.x

[46] Al-Salmani A, Juma T, Al-Noobi A, (2015) Characterization of depression among patients at urban primary healthcare centers in Oman. Int J Psychiatry Med 49, 1–18. 25838317 10.2190/PM.49.1.a

[47] Al-Amer RM, Sobeh MM, Zayed AA, (2011) Depression among adults with diabetes in Jordan: risk factors and relationship to blood sugar control. J Diabetes Complications 25, 247–252. 21601482 10.1016/j.jdiacomp.2011.03.001

[48] Emeka PM, Mukalaf AA, Helal HA, (2017) Prevalence of poor glycemic and blood pressure control and pattern of drug use among primary health-care outpatients in Al Ahsa Saudi Arabia. Int J Health Sci (Qassim) 11, 38–44. 28936150 PMC5604263

[49] Ashur ST, Shah SA, Bosseri S, (2016) Glycaemic control status among type 2 diabetic patients and the role of their diabetes coping behaviours: a clinic-based study in Tripoli, Libya. Libyan J Med 11, 31086–31086. 27005896 10.3402/ljm.v11.31086 PMC4803895

[50] Ali MK, Bullard KM, Imperatore G, (2012) Characteristics associated with poor glycemic control among adults with self-reported diagnosed diabetes — national health and nutrition examination survey, United States, 2007–2010. MMWR Morb Mortal Wkly Rep 61S, 32–37. 22695461

[51] Moursi M (2009) The 2004 National Diet and Nutrition Survey of the Sultanate of Oman-Preliminary Report. Ministry of Health.

[52] World Health Organization (2020) Sultanate of Oman STEPS Survey 2017: Fact Sheet Omani and Non-Omani 18+. https://www.who.int/ncds/surveillance/steps/oman/en/ (accessed November 2020).

[53] Academy of Nutrition and Dietetics Evidence Analysis Library (2019) Recommendations summary: Medical Nutrition Therapy (2015). Diabetes Type 1 and 2 Diabetes (DM) Guideline (2015). https://www.andeal.org/template.cfm?template=guide_summary&key=4494S (accessed April 2019).

[54] Rosal MC, Ockene IS, Restrepo A, (2011) Randomized trial of a literacy-sensitive, culturally tailored diabetes self-management intervention for low-income Latinos. Diabetes Care 34, 838. 21378213 10.2337/dc10-1981 PMC3064037

[55] Paschalides C, Wearden AJ, Dunkerley R, (2004) The associations of anxiety, depression and personal illness representations with glycaemic control and health-related quality of life in patients with type 2 diabetes mellitus. J Psychosom Res 57, 557–564. 15596162 10.1016/j.jpsychores.2004.03.006

[56] Al-Sinani M, Min Y, Ghebremeskel K, (2010) effectiveness of and adherence to dietary and lifestyle counselling: effect on metabolic control in type 2 diabetic Omani patients. Sultan Qaboos Univ Med J 10, 341–349. 21509254 PMC3074742

[57] Ridgeway NA, Harvill DR, Harvill LM, (1999) Improved control of type 2 diabetes mellitus: a practical education/behavior modification program in a primary care clinic. South Med J 92, 667–672. 10414474 10.1097/00007611-199907000-00004

[58] Sainsbury E, Kizirian NV, Partridge SR, (2018) Effect of dietary carbohydrate restriction on glycemic control in adults with diabetes: a systematic review and meta-analysis. Diabetes Res Clin Pract 139, 239–252. 29522789 10.1016/j.diabres.2018.02.026

[59] Esposito K, Maiorino MI, Ceriello A, (2010) Review: Prevention and control of type 2 diabetes by Mediterranean diet: a systematic review. Diabetes Res Clin Pract Suppl 89, 97–102. 10.1016/j.diabres.2010.04.019 20546959

[60] Collins MM, Corcoran P & Perry IJ (2009) Anxiety and depression symptoms in patients with diabetes. Diabetic Med 26, 153–161. 19236618 10.1111/j.1464-5491.2008.02648.x

[61] Yekta Z, Pourali R & Yavarian R (2010) Behavioural and clinical factors associated with depression among individuals with diabetes. East Mediterr Health J 16, 286–291. 20795442

[62] Yu R, Y-Hua L & Hong L (2010) Depression in newly diagnosed type 2 diabetes. Int J Diabetes Dev Ctries 30, 102–104. 20535315 10.4103/0973-3930.62601 PMC2878688

[63] Katon W, Von Korff M, Ciechanowski P, (2004) Behavioral and clinical factors associated with depression among individuals with diabetes. Diabetes Care 27, 914. 15047648 10.2337/diacare.27.4.914

[64] Hall PA, Rodin GM, Vallis TM, (2009) The consequences of anxious temperament for disease detection, self-management behavior, and quality of life in Type 2 diabetes mellitus. J Psychosom Res 67, 297–305. 19773022 10.1016/j.jpsychores.2009.05.015

